# Chest physiotherapy improves lung aeration in hypersecretive critically ill patients: a pilot randomized physiological study

**DOI:** 10.1186/s13054-020-03198-6

**Published:** 2020-08-03

**Authors:** Federico Longhini, Andrea Bruni, Eugenio Garofalo, Chiara Ronco, Andrea Gusmano, Gianmaria Cammarota, Laura Pasin, Pamela Frigerio, Davide Chiumello, Paolo Navalesi

**Affiliations:** 1 Anesthesia and Intensive Care Unit, Department of Medical and Surgical Sciences, University Hospital Mater Domini, Magna Graecia University, Catanzaro, Italy; 2 Anesthesia and Intensive Care, Sant’Andrea Hospital, ASL VC, Vercelli, Italy; 3 Department of Anesthesia and Intensive Care, “Maggiore della carità” University Hospital, Novara, Italy; 4 Department of Anesthesia and Intensive Care, Azienda Ospedaliera-Università di Padova, Padua, Italy; 5 ASST Grande Ospedale Metropolitano Niguarda, Milan, Italy; 6 SC Anestesia e Rianimazione, Ospedale San Paolo - Polo Universitario, ASST Santi Paolo e Carlo, Milan, Italy; 7 Dipartimento di Scienze della Salute, Università degli Studi di Milano, Milan, Italy; 8 Centro Ricerca Coordinata di Insufficienza Respiratoria, Università degli Studi di Milano, Milan, Italy; 9 Dipartimento di Medicina-DIMED, Università degli Studi di Padova, Via Giustiniani, 2 –, 35128 Padova, Italy

**Keywords:** Cough, Mechanical ventilation, Acute respiratory failure, High-frequency chest wall oscillation, Chest physiotherapy, Lung aeration, Electrical impedance tomography

## Abstract

**Background:**

Besides airway suctioning, patients undergoing invasive mechanical ventilation (iMV) benefit of different combinations of chest physiotherapy techniques, to improve mucus removal. To date, little is known about the clearance effects of oscillating devices on patients with acute respiratory failure undergoing iMV. This study aimed to assess (1) the effects of high-frequency chest wall oscillation (HFCWO) on lung aeration and ventilation distribution, as assessed by electrical impedance tomography (EIT), and (2) the effect of the association of HFCWO with recruitment manoeuvres (RM).

**Methods:**

Sixty critically ill patients, 30 classified as normosecretive and 30 as hypersecretive, who received ≥ 48 h of iMV, underwent HFCWO; patients from both subgroups were randomized to receive RM or not, according to two separated randomization sequences. We therefore obtained four arms of 15 patients each. After baseline record (T0), HFCWO was applied for 10 min. At the end of the treatment (T1) or after 1 (T2) and 3 h (T3), EIT data were recorded. At the beginning of each step, closed tracheobronchial suctioning was performed. In the RM subgroup, tracheobronchial suctioning was followed by application of 30 cmH_2_O to the patient’s airway for 30 s. At each step, we assessed the change in end-expiratory lung impedance (ΔEELI) and in tidal impedance variation (ΔTIV), and the center of gravity (COG) through EIT. We also analysed arterial blood gases (ABGs).

**Results:**

ΔTIV and COG did not differ between normosecretive and hypersecretive patients. Compared to T0, ΔEELI significantly increased in hypersecretive patients at T2 and T3, irrespective of the RM; on the contrary, no differences were observed in normosecretive patients. No differences of ABGs were recorded.

**Conclusions:**

In hypersecretive patients, HFCWO significantly improved aeration of the dorsal lung region, without affecting ABGs. The application of RM did not provide any further improvements.

**Trial registration:**

Prospectively registered at the Australian New Zealand Clinical Trial Registry (www.anzctr.org.au; number of registration: ACTRN12615001257550; date of registration: 17th November 2015).

## Background

Mucociliary clearance of secretion is the primary innate protective mechanism of the respiratory tract to remove inhaled particles and microorganism from the tracheobronchial system; it depends from the interaction between ciliated columnar cells and the viscoelastic properties of bronchial secretions [1].

The presence of an endotracheal tube impairs the bronchial mucus velocity transport in anaesthetized dogs [2]; in critically ill patients undergoing invasive mechanical ventilation (iMV), it seriously impairs cough reflex and mucociliary escalator function [3, 4], promoting the accumulation of tracheobronchial secretions, leading to sequestration and densification of secretions in the lower airways and increasing the risk of pneumonia [5] and lung atelectasis [6].

Usual care of the intubated patient includes direct suction applied through the endotracheal tube, which clears a small portion of the airway, is ineffective for clearing secretions in the peripheral airways, and leaves the patient dependent on mucociliary clearance rather than on cough clearance [7].

In intubated patients, the application of various combinations of chest physiotherapy techniques is a commonly used intensive care procedure [8]. There is supportive evidence that various combinations of chest physiotherapy assist in the re-expansion of atelectatic lung [9], confer short-term improvement in total lung-thorax compliance [10] and expiratory flow rates [11], and reduce the incidence of ventilator-associated pneumonia [12].

Oscillating devices have been repeatedly shown to improve mucus clearance in patients with chronic retention of airway secretions such as cystic fibrosis [13–15]. On the opposite, little is known about their effects on patients with acute respiratory failure (ARF) undergoing iMV.

High-frequency chest wall oscillation (HFCWO) is an airway clearance technique by which external forces are applied to the chest through an inflatable wrap connected to a device that generates vibrations at variable frequency and intensity.

Various mechanisms of action have been proposed for HFCWO: (1) augmentation of the expiratory flow, which increases the annular flow of mucus towards the oropharynx when exceeding by more than 10% the inspiratory peak flow [16–18]; (2) improvement of mucus rheological (spinnability and viscoelastic) properties [19, 20]; (3) increase of ciliary motility by reflected stimulation of vagus nerve, in particular when the applied oscillations range between 11 and 15 Hz [21]; and (4) enhancement of the gas-liquid transport [16, 22]. Recent findings showed that HFCWO, when compared to conventional physiotherapy, improves the amount of aerated lung regions and oxygenation of intubated patients in intensive care unit (ICU) [23, 24].

Electrical impedance tomography (EIT) has been introduced in ICU clinical practice as a non-invasive bedside monitoring system able to evaluate the aeration and ventilation of different lung regions [25]. This technique is based on the injection of small currents and voltage measurements through multiple electrodes placed around the chest of the patient. The recorded currents generate a cross-sectional image representing the change of impedance throughout the patient’s respiration and, therefore, the lung ventilation [25].

The aim of this pilot randomized physiological study is assessing the effects of HFCWO on lung aeration and ventilation distribution, as assessed by EIT, in normosecretive and hypersecretive mechanically ventilated patients. In addition, we also evaluate the association of HFCWO with recruitment manoeuvres (RM).

## Methods

### Study design

This four-arms randomized study was conducted in the Sant’Andrea Hospital of Vercelli (Italy) after local Ethical committee approval (Ethical Committee Approval number AslVc.Rian.14.03, on 11th September 2014). It included patients who required more than 48 h of iMV and who satisfied eligibility criteria. All enrolled patients underwent HFCWO and were randomized to receive a RM or not.

The primary outcome of the study was the variation in end-expiratory lung impedance. Secondary outcomes were the tidal impedance and arterial partial pressure of oxygen (PaO_2_) variations among groups. The following variables were collected: socio-demographic characteristics, body mass index (BMI), comorbidities, reason for ICU admission, Acute Physiology and Chronic Health Disease Classification System II score (APACHEII), Sequential Organ Failure Assessment (SOFA), and Simplified Acute Physiology Score (SAPS2). Changes in vital parameters and arterial blood gas analysis (ABGs) were also recorded.

All participants provided written informed consent. This trial followed the Consolidated Standards of Reporting Trials (CONSORT) reporting guidelines and was prospectively registered in the Australian New Zealand Clinical Trial Registry (ACTRN12615001257550; www.anzctr.org.au, date of registration 17th November 2015).

### Subjects

We considered eligible any patient ≥ 18 years who was admitted to the ICU and received more than 48 h of iMV with heated humidification. Exclusion criteria were as follows: (1) life-threatening cardiac arrhythmias or signs of ischaemia, (2) pneumothorax, (3) haemoptysis, (4) acute spinal injury, (5) need for vasopressors, (6) brain injury, (7) pulmonary embolism, (8) recent chest trauma or burn, (9) recent surgery, (10) pregnancy, (11) enrolment in other research protocols, and (12) denied consent.

Eligible patients were classified as “normosecretive” or “hypersecretive”. Definition of “normosecretive” or “hypersecretive” was based on the criteria adopted in previously published studies [26–29]. Briefly, patients who required two or more broncoaspirations/hour in the previous 8 h were considered “hypersecretive”.

**Fig. 1:**
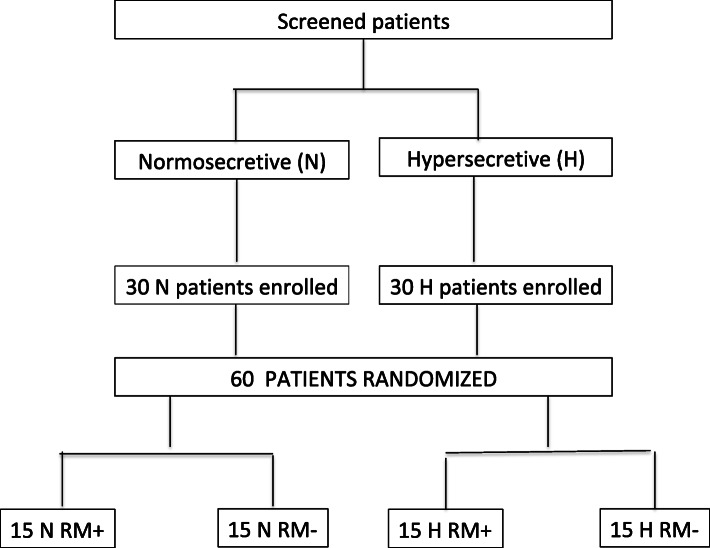
Flowchart of the study. The figure depicts the study flowchart, which includes four arms

Suction occurred only if auscultation revealed secretions in the larger airway, if the airway pressure waveform indicated fluid in the system and/or the peak airway pressures increased [30]. An equal number of patients were enrolled from the hypersecretive and normosecretive group. Two separate randomization sequences were computer-generated (one for each subpopulation) and sealed in opaque numbered envelopes. In practice, a total of 60 patients were randomized in this four-arms pilot physiological study. Among them, 30 patients belonged to the normosecretive group and 30 patients to the hypersecretive one. Each patient was randomized to receive RM or not. Therefore, we obtained four groups of 15 patients: normosecretive not receiving RM (N RM−), normosecretive receiving RM (N RM+), hypersecretive not receiving RM (H RM−), and hypersecretive receiving RM (H RM+) (Fig. 1).

### Study protocol and data acquisition

After randomization, one silicon EIT belt of proper size with 16 electrodes was placed around the patient’s chest between the 4th and 6th intercostal space and connected to an EIT device (PulmoVista 500; Draeger Medical GmbH, Lübeck, Germany) [25, 31, 32]. Quality of signal was ascertained through a dedicated tool of the EIT device. EIT belt positioning was therefore marked on the skin, in order to avoid its displacement during the whole study period. Patients were switched to receive iMV by a ventilator connected to the EIT device through a RS232 interface (V500; Draeger Medical GmbH, Lübeck, Germany); a heated humidifier (MR850 heated humidifier; Fisher & Paykel, Auckland, New Zealand) was also used. Ventilatory mode and settings were those clinically indicated by the attending physicians, while heated humidifier was set at 37 °C and relative humidity at 100% (i.e. 44 mg/L of absolute humidity). Ventilator settings and analgo-sedation regimen were not modified throughout the entire study protocol and no supportive measures such as instillation of isotonic or hypertonic saline were used.

Subsequently, a dedicated inflatable wrap garment of proper size was placed around the patient’s chest and connected to a generator of pressure and oscillation (The Vest 105 System, Hill Rom, St. Paul, MN, USA). Finally, tracheobronchial suctioning was assured throughout a closed aspiration system (KimVent, Turbo-Cleaning Closed Suction System, Kimberly-Clark, Roswell, GA, USA). After EIT baseline record (T0), HFCWO was applied for 10 min with an oscillation frequency of 12 Hz [33, 34]. Soon after the end of the treatment (T1) or after 1 (T2) and 3 h (T3), EIT data were recorded for 10 min. At the beginning of each step of the protocol, closed tracheobronchial suctioning was performed. In the subgroup of patients randomized to receive RM, tracheobronchial suctioning was followed by application of 30 cmH_2_O to the patient’s airway for 30 s.

Predefined criteria for protocol interruption were as follows: (1) onset of haemodynamic instability, (2) life-threatening arrhythmias or electrocardiographic signs of ischaemia, and (3) worsening of oxygen saturation (SatO_2_), as assessed by pulse oxymetry.

### Data analysis

Detailed information about data acquisition and analysis is provided in the Supplemental Material (Supplemental Material). Briefly, we computed the tidal impedance variation (TIV) as the difference of impedance between the end of inspiration and expiration [31], and changes in TIV (ΔTIV, mL) and in end-expiratory lung impedance (ΔEELI, mL), which is a surrogated measure of the end-expiratory lung volume [31, 32, 35]. We also defined two contiguous regions of interest (ROIs) of the same size (ventral and dorsal) and computed TIV, ΔTIV, and ΔEELI for both [32, 35]. The amount of pixel of non-ventilated area was also calculated and expressed as percentage of the pixel number. We also computed the centre of gravity (COG), which expresses the distribution of TIV in ventral to dorsal direction, calculated by dividing the dorsal by the overall TIV, expressed as percentage [36].

### Statistical analysis

Because of the lack of previous similar studies and given the descriptive and physiologic purpose of our pilot randomized study, we arbitrarily decided to enrol a sample of 60 patients, 30 for each subpopulation (i.e. normosecretive and hypersecretive patients). Normally distributed continuous data were described as means and standard deviation (SD) and non-normal distributed data were described as median and interquartile range [25th–75th interquartile range]. Normality of continuous data was assessed through the Kolmogorov-Smirnov test. Comparisons between groups were performed by using the Student *t* test or the Mann-Whitney *U* test, for the continuous variables normally or non-normally distributed, respectively. Categorical variables were reported as numbers and percentages. Categorical data were compared with a chi-square test or Fisher test, when required. To assess the effects of the “amount of secretions”, “application of a RM” and their interaction with the “time after HFCWO application”, continuous variables were analysed with the analysis of variance (ANOVA) or Friedman test, according to the Gaussian distribution of data. Furthermore, positive end-expiratory pressure (PEEP) values between the four groups were compared through a one-way ANOVA test. Post hoc Bonferroni test was applied for pair-wise multiple comparisons, when indicated.

We considered significant two-sided *p* values < 0.05. Statistical analysis was performed using the Sigmaplot v. 12.0 (Systat Software Inc., San Jose, CA).

## Results

Thirty normosecretive and 30 hypersecretive patients were enrolled between December 2015 and June 2016. All patients completed the study protocol without any complication and were included in data analysis. Demographic, anthropometric, and clinical characteristics of included patients are presented in Table 1.

**Table 1:** Differences in clinical characteristics at ICU admission between hypersecretive and normosecretive patients

	**All (*n*** **= 60)**	**Normosecretive (*n*** **= 30)**	**Hypersecretive (*n*** **= 30)**	*p*value*
**Age, median (IQR)**	69 (59–78)	69 (61–76)	67 (58–78)	0.70
**Gender (male)**	35 (58.33%)	17 (56.7%)	18 (60%)	0.79
**BMI, median (IQR)**	27.19 (24.22–29.39)	26.79 (24.10–29.38)	27.36 (24.22–29.40)	0.62
**Height, cm; median (IQR)**	170 (165–175)	169 (165–175)	170 (170–175)	0.39
**Comorbidities,*n***				
Ischaemic heart disease	6 (10.00%)	4 (13.30%)	2 (6.7%)	0.67
Congestive heart failure	12 (20.00%)	7 (23.30%)	5 (16.70%)	0.52
Hypertension	30 (50.00%)	15 (50%)	15 (50%)	1.00
Cerebrovascular events	12 (20.00%)	6 (20.00%)	6 (20.00%)	1.00
Cognitive impairment	2 (3.33%)	1 (3.33%)	1 (3.33%)	1.00
COPD	20 (33.33%)	10 (33.33%)	10 (33.33%)	1.00
Diabetes mellitus	17 (28.33%)	8 (26.70%)	9 (30.00%)	0.77
Moderate to severe CKD	4 (6.67%)	1 (3.30%)	3 (10.00%)	0.61
**Cause of admission,*n***				
Respiratory	23.00 (38.33%)	11 (36.70%)	12 (40.00%)	0.96
Cardiovascular	9.00 (15.00%)	5 (16.70%)	4 (13.30%)	1.00
Neurologic	19.00 (31.67)	10 (33.30%)	9 (30.00%)	0.96
Trauma/burn/other	9.00 (15.00%)	4 (13.30%)	5 (16.7%)	1.00
**APACHE-II median (IQR)**	17.50 (14.00–23.00)	16.00 (12.00–24.00)	19.00 (15.00–22.00)	0.51
**SOFA, median (IQR)**	7.50 (6.00–12.00)	6.00 (4.5–11.25)	8.5 (6.0–11.75)	0.18
**SAPS2, mean (SD)**	53.05 (18.73)	53.47 (21.37)	52.63 (16.02)	0.86
**Mode of ventilation (VC/PSV),*n***	8/52	4/26	4/26	1.00

*p value for the comparison between normosecretive and hypersecretive patients

*BMI* body mass index, *COPD* chronic obstructive pulmonary disease, *CKD* chronic kidney disease, *APACHE II* Acute Physiology and Chronic Health Disease Classification System II, *SOFA* Sequential Organ Failure Assessment, *SAPS2* Simplified Acute Physiology Score, *VC* volume controlled, *PSV* pressure support ventilation

**Fig. 2:**
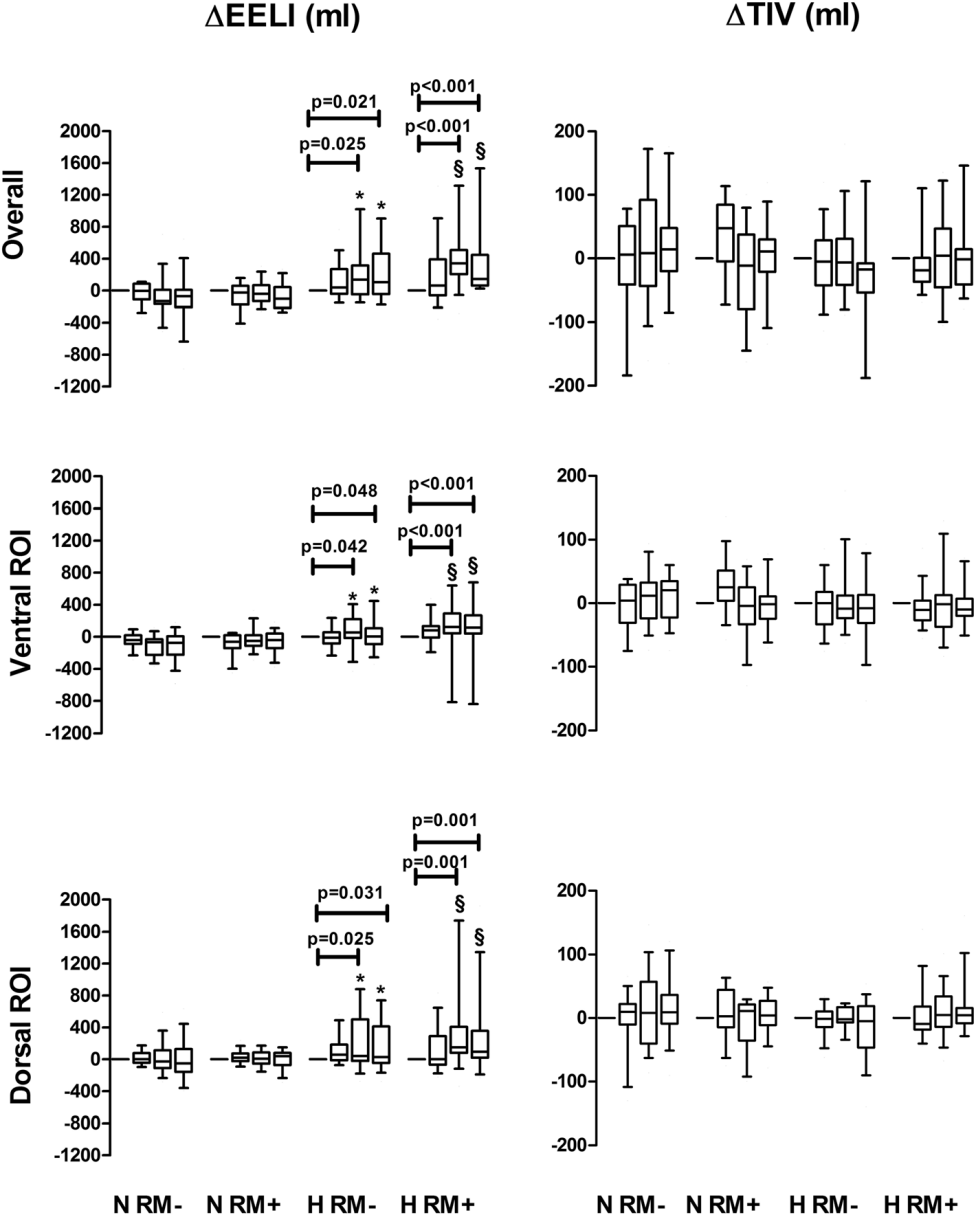
Changes of electrical impedance tomography values in the four study arms. Box plots of changes of end-expiratory lung impedance (DEELI) and tidal impedance variation (DTIV) are depicted in the four study arms, at baseline (T0), squared at T1, triangles at T2, and rhombus at T3. The bottom and top of the box indicate the 25th and 75th percentile, the horizontal band near the middle of the box is the median, and the ends of the whiskers represent the 10th and 90th percentiles. Statistically significant *p* values within study arms are reported in the figures. *p < 0.05, compared to N RM− at the same time point; ^§^*p* < 0.05, compared to N RM+ at the same time point

No differences in percentage of not ventilated areas, TIV, ΔTIV, and centre of gravity were found between normosecretive and hypersecretive patients at all time points. Hypersecretive patients showed higher ΔEELI at T2 and T3, as opposed to the respective time points in normosecretive patients (Table 2; Fig. 2).

**Table 2:** Evaluation of the effect of HFCWO on subpopulation (normosecretive vs hypersecretive) along time

	**Normosecretive (*n*** **= 30)**				**Hypersecretive (*n*** **= 30)**				
	**T0**	**T1**	**T2**	**T3**	**T0**	**T1**	**T2**	**T3**	***p*value**
Not ventilated area (%)	0.2 [0.0; 2.9]	0.0 [0.0; 2.5]	0.0 [0.0; 1.8]	0.0 [0.0; 1.9]	0.6 [0.0; 2.7]	1.2 [0.0; 4.3]	0.4 [0.0; 3.7]	1.3 [0.0; 4.9]	0.082
TIV (mL)	444 [345; 539]	454 [372; 524]	447 [374; 536]	453 [358; 547]	474 [364; 540]	442 [363; 551]	461 [363; 555]	458 [363; 542]	0.123
Dorsal	186 [142; 248]	188 [131; 259]	201 [132; 246]	210 [119; 252]	174 [143; 229]	161 [133; 224]	179 [145; 223]	169 [134; 221]	0.771
Ventral	222 [169; 325]	248 [180; 319]	242 [185; 310]	234 [192; 307]	291 [212; 351]	285 [209; 360]	278 [197; 361]	281 [204; 336]	0.150
ΔTIV (mL)	0 [0; 0]	31 [− 28; 76]	− 7 [− 63; 65]	11 [− 20; 34]	0 [0; 0]	− 8 [− 38; 14]	− 3 [− 43; 34]	− 11 [− 44; 6]	0.117
Dorsal	0 [0; 0]	9 [− 11; 31]	9 [− 37; 28]	7 [− 10; 27]	0 [0; 0]	− 3 [− 15; 12]	4 [− 12; 17]	2 [− 28; 17]	0.408
Ventral	0 [0; 0]	7 [− 15; 37]	9 [− 28; 30]	7 [− 23; 28]	0 [0; 0]	− 4 [− 28; 9]	− 5 [− 36; 12]	− 9 [− 21; 8]	0.204
Centre of gravity (%)	51.9 [48.9; 54.6]	52.2 [49.8; 54.3]	51.4 [49.9; 54.0]	51.8 [48.6; 53.8]	54.8 [51.7; 57.1]	54.9 [51.6; 57.2]	54.6 [52.3; 56.9]	54.6 [51.5; 56.8]	0.054
ΔEELI (mL)	0 [0; 0]	− 22 [− 144; 73]	− 167 [− 47; 36]	− 98 [− 207; 30]	0 [0; 0]	42 [− 46; 316]	269 [92; 464]*^,a^	131 [34; 453]*^,b^	< 0.001
Dorsal	0 [0; 0]	9 [− 39; 75]	− 6 [− 85; 94]	− 13 [− 146; 94]	0 [0; 0]	11 [− 44; 231]	97 [15; 431]*^,a^	67 [11; 373]*^,b^	< 0.001
Ventral	0 [0; 0]	− 39 [− 134; 20]	−61 [−128; 12]	− 62 [− 188; 29]	0 [0; 0]	41 [− 60; 117]	115 [7; 238]*	59 [− 63; 194]*	< 0.001
Heart rate (beat/min)	84 (16)	85 (18)	84 (20)	82 (18)	80 (14)	80 (14)	77 (14)	75 (13)	0.916
Mean arterial pressure (mmHg)	86 (14)	86 (15)	85 (14)	85 (14)	88 (12)	88 (13)	85 (11)	83 (11)	0.811
Respiratory rate (breath/min)	18 (4)	18 (4)	18 (4)	19 (4)	18 (6)	19 (6)	19 (6)	18 (6)	0.954
pH	7.44 (0.06)	7.42 (0.06)	7.43 (0.06)	7.43 (0.06)	7.41 (0.06)	7.41 (0.06)	7.42 (0.05)	7.42 (0.05)	0.967
PaCO_2_ (mmHg)	40.9 (8.1)	42.2 (8.1)	41.7 (8.1)	41.8 (7.8)	44.3 (8.6)	44.3 (9.4)	43.7 (8.3)	43.4 (8.3)	0.952
PaO_2_/FiO_2_ (mmHg)	238 (75)	234 (65)	241 (61)	239 (60)	214 (56)	219 (59)	222 (55)	220 (52)	0.973

*T0* baseline assessment, *T1* assessment soon after the end of the treatment, *T2* assessment 1 h after the end of the treatment, *T3* assessment 3 h after the end of the treatment, *TIV* tidal impedance variation, *ΔTIV* difference of TIV from T0, *ΔEELI* difference of end-expiratory lung impedance from T0, *PaCO*_*2*_ arterial partial pressure of carbon dioxide, *PaO*_*2*_*/FiO*_*2*_ ratio between arterial partial pressure and inspired fraction of oxygen

*p < 0.05 normosecretive vs. hypersecretive within the same time point

^a^*p* < 0.05 T0 vs. T2 within the same subpopulation

^b^*p* < 0.05 T0 vs. T3 within the same subpopulation. All data are expressed as mean (standard deviation)

EIT parameters did not differ between patients randomized to receive or not RM (Table 3). When compared to N RM− patients, the H RM− group showed higher overall dorsal and ventral ΔEELI. Similarly, both overall dorsal and ventral ΔEELI values were higher in H RM+, as compared to N RM+. On the contrary, no differences in ΔTIV were recorded. (Table E1, supplemental material).

**Table 3:** Evaluation of the effect of HFCWO on two subgroups of patients receiving or not a RM

	**Not receiving RM (*n*** **= 30)**				**Receiving RM (*n*** **= 30)**				
	**T0**	**T1**	**T2**	**T3**	**T0**	**T1**	**T2**	**T3**	***p*value**
Not ventilated area (%)	0.2 [0.0; 2.8]	1.1 [0.0; 3.9]	0.4 [0.0; 4.2]	0.9 [0.0; 4.2]	0.6 [0.0; 2.8]	0.3 [0.0; 2.5]	0.0 [0.0; 3.0]	0.6 [0.0; 3.0]	0.893
TIV (mL)	446 [336; 488]	429 [361; 497]	433 [357; 509]	440 [345; 497]	473 [366; 607]	472 [414; 629]	471 [382; 578]	490 [67; 563]	0.111
Dorsal	158 [138; 222]	158 [121; 214]	161 [133; 217]	157 [119; 240]	193 [160; 247]	210 [156; 266]	205 [169; 243]	209 [174; 240]	0.122
Ventral	223 [180; 313]	246 [177; 313]	253 [185; 322]	235 [200; 321]	286 [196; 353]	303 [202; 362]	259 [197; 335]	270 [203; 335]	0.679
ΔTIV (mL)	0 [0; 0]	− 4 [− 41; 42]	− 4 [− 42; 47]	− 9 [− 26; 34]	0 [0; 0]	− 2 [34; 66]	− 3 [− 53; 40]	1 [− 23; 27]	0.995
Dorsal	0 [0; 0]	6 [− 11; 21]	1 [− 23; 20]	4 [− 28; 20]	0 [0; 0]	− 4 [− 16; 29]	6 [− 20; 23]	4 [− 10; 18]	0.906
Ventral	0 [0; 0]	1 [− 31; 21]	2 [− 24; 28]	− 6 [− 24; 28]	0 [0; 0]	4 [− 20; 31]	− 3 [− 36; 15]	− 4 [− 21; 9]	0.932
Centre of gravity (%)	53.8 [50.8; 56.0]	53.5 [50.8; 56.9]	53.7 [50.2; 57.5]	54.0 [50.3; 56.8]	53.3 [49.6; 54.9]	53.2 [50.0; 55.4]	52.8 [50.2; 54.8]	52.3 [50.1; 54.5]	0.561
ΔEELI (mL)	0 [0; 0]	29 [−59; 108]	0 [−138; 225]	1 [−170; 162]	0 [0; 0]	2 [−95; 153]	126 [−45; 352]	61 [−102; 169]	0.272
Dorsal	0 [0; 0]	9 [− 30; 125]	6 [− 77; 188]	7 [− 83; 277]	0 [0; 0]	7 [− 51; 140]	88 [− 12; 189]	48 [− 12; 154]	0.069
Ventral	0 [0; 0]	− 25 [− 84; 40]	− 22 [− 128; 109]	− 23 [− 122; 59]	0 [0; 0]	21 [− 116; 86]	32 [− 94; 175]	42 [− 104; 131]	0.413
Heart rate (beat/min)	79 (14)	81 (16)	79 (18)	77 (16)	84 (16)	84 (16)	82 (17)	80 (16)	0.983
Mean arterial pressure (mmHg)	84 (13)	84 (12)	83 (12)	83 (11)	90 (13)	90 (15)	87 (13)	85 (13)	0.830
Respiratory rate (breath/min)	18 (5)	18 (5)	17 (5)	17 (5)	19 (5)	19 (5)	19 (5)	19 (4)	0.995
pH	7.42 (0.06)	7.42 (0.06)	7.43 (0.06)	7.43 (0.06)	7.42 (0.06)	7.41 (0.05)	7.42 (0.05)	7.42 (0.05)	0.972
PaCO_2_ (mmHg)	43.4 (8.5)	43.4 (8.2)	43.7 (8.6)	43.8 (8.3)	41.8 (8.4)	43.1 (9.3)	41.7 (7.8)	41.5 (7.7)	0.926
PaO_2_/FiO_2_ (mmHg)	240 (73)	236 (66)	242 (61)	241 (62)	212 (58)	217 (57)	221 (54)	218 (49)	0.977

*RM* recruiting manoeuvre, *T0* baseline assessment, *T1* assessment soon after the end of the treatment, *T2* assessment 1 h after the end of the treatment, *T3* assessment 3 h after the end of the treatment, *TIV* tidal impedance variation, *ΔTIV* difference of TIV from T0, *ΔEELI* difference of end-expiratory lung impedance from T0, *PaCO*_*2*_ arterial partial pressure of carbon dioxide, *PaO*_*2*_*/FiO*_*2*_ ratio between arterial partial pressure and inspired fraction of oxygen

Vital parameters and ABGs were not different between any subgroup.

## Discussion

Our study shows that chest physiotherapy by HFCWO may improve lung aeration of hypersecretive mechanically ventilated patients, without affecting gas exchange. On the contrary, it does not produce any significant changes in normosecretive patients. In addition, our study shows that the application of RM does not add any further advantage, both in normosecretive and hypersecretive groups of patients.

In the last years, mechanical assisted cough devices have been increasingly used in the acute setting; however, these devices aim to remove secretions from the large airways to the airway opening, through a sequential application of positive and negative (sub-atmospheric) pressure to the airway [37]. Differently, HFCWO aims to mobilize secretions from the smallest and deepest airways towards the upper and larger airways.

To date, only a few data exist on the application of HFCWO in mechanically ventilated patients. In fact, most of the published studies mainly refer to the application of this technique in different populations. Esguerra-Gonzales et al. proved the efficacy of HFCWO in oxygenation improvement in 45 non-intubated recipients of lung transplantation, as compared to the conventional physiotherapy treatment [38]. Accordingly, Kuyrukluyildiz et al. showed that the application of HFCWO in intubated ICU patients provided more secretion mobilization, translating in better oxygenation after 72 h of repeated applications [23]. On the contrary, Clinkscale et al. did not find any benefit in hypersecretive patients treated with HFCWO, although this latter was better tolerated by patients than conventional physiotherapy [39]. Similar results were reported in patients with chronic obstructive pulmonary disease [40], neuromuscular disorders [41–43], and after lung resection surgery [44].

Since mucus retention can occlude distal and smaller airways, inducing atelectasis of the alveoli [45], we decided to randomize some normosecretive and hypersecretive patients to receive a RM after HFCWO and closed suction. In fact, after application of HFCWO and suctioning, sputum and airway obstruction were supposed to be removed, while distal and smaller airways to be reopened. Furthermore, suctioning may induce a loss of end-expiratory lung volume and atelectasis [46]. Therefore, the application of RMs should have reopened the lung, which was subsequently kept open by positive pressures of mechanical ventilation. For these reasons, we decided to investigate if the additional application of a RM after closed suctioning may have provided further advantages to the patient.

The findings of the present study are of potential clinical interest. First, our results suggest that during mechanical ventilation, HFCWO should be applied only in hypersecretive patients. This is important in order to save time and workload for nurses and/or physiotherapists. Furthermore, we observed a significant increment of ΔEELI, which is a surrogated measure for the functional residual capacity. In principle, lung recruitment, increasing functional residual capacity, should be coupled with improved oxygenation [23, 31]. Surprisingly, we did not observe such an improvement. These findings could be explained by a correction of the ventilation-perfusion mismatch, rather than a reduction of the intrapulmonary shunt, as also indirectly confirmed by the lack of effect produced by RM. In this manner, the oxygenation enhancement would be masked/covered/obscured by oxygen administration, which occurred in all patients (mean FiO_2_ 0.40 ± 0.09 in the hypersecretive group). Secondarily, since the physiological benefits we observed tended to reduce after 3 h from the application of HFCWO, we might hypothesize that the treatment should be repeated every 4 h. However, further studies are necessary to support this hypothesis.

Before drawing our conclusions, some limitations deserve discussion. First, we arbitrarily defined normosecretive and hypersecretive patients according to the number of tracheal-bronchial aspiration per hour in the prior 8 h, which is definitely a gross and simplistic criterion. The same criterion was adopted in previously published studies [26–28] and, worth remarking, allowed us to differentiate two subpopulations with different physiologic outcomes. In another previous study, a single operator assessed the amount of secretions, who classified patients as producing either no, mild, moderate, or abundant endotracheal secretions based on personal observations in the preceding 4 to 6 h [29]. However, this method is quite subjective and opened to criticisms. In addition, no standardized bronchoaspiration protocol was applied and nurse subjectivity might have generated a bias in patients’ selection. Moreover, the number of patients enrolled was small. Worth remarking, however, several studies aimed to assess modifications in EIT parameters in response to different interventions enrolled similar, or even lower, number of patients [26, 28, 41, 42]. Although the great effect size we observed suggests the appropriateness of the sample, our study has to be considered as hypothesis generating. In addition, this is a physiologic study and we cannot provide any information about the clinical implications of our finding. Knowledge of pathophysiologic mechanisms and physiologic consequences of any intervention are fundamental for designing meaningful randomized, controlled trials. Finally, the vast majority of patients were ventilated in pressure support ventilation, which is an assisted modality. Although ventilator settings were not modified throughout the entire study protocol, the tidal volume might have been changed. In fact, not only the inspiratory airway pressure participates in the tidal volume generation, but also respiratory muscle activation and respiratory mechanics [47, 48]. In the attempt to optimize the control of these variables, we also kept the analgo-sedation regimen unmodified, since analgesics and sedatives may influence the respiratory drive and/or pattern [49–51]. As a result, ΔTIV did not change throughout the study protocol, either between normosecretive and hypersecretive patients or between patients randomized to receive or not a RM.

## Conclusions

Chest physiotherapy by HFCWO significantly improves aeration of the dorsal region of the lung in hypersecretive mechanically ventilated patients without affecting gas exchange. The application of RM does not add any further benefit. HFCWO did not produce any effect in normosecretive patients.

## Supplementary information

**Additional file 1: Table E1.** Data from the 4 subgroups of patients stratified according the amount of secretions and application of recruiting manoeuvres.

## Data Availability

The authors will share all of the individual participant data collected during the trial after de-identification, to researchers who provide a methodologically sound proposal. The full protocol and raw data are available at longhini.federico@gmail.com

## Abbreviations

ABGs: Arterial blood gas analysis
APACHE-II: Acute Physiology and Chronic Health Disease Classification System II score
ARF: Acute respiratory failure
BMI: Body mass index
CKD: Chronic kidney disease
COG: Centre of gravity
COPD: Chronic obstructive pulmonary disease
ΔTIV: Changes in tidal impedance variation
ΔEELI: Changes in end-expiratory lung impedance
EIT: Electrical impedance tomography
HFCWO: High-frequency chest wall oscillation
H RM+: Hypersecretive receiving recruitment manoeuvres
H RM: Hypersecretive not receiving recruitment manoeuvres
N RM+: Normosecretive receiving recruitment manoeuvres
N RM: Normosecretive not receiving recruitment manoeuvres
ICU: Intensive care unit
iMV: Invasive mechanical ventilation
PaO_2_: Arterial partial pressure of oxygen
PEEP: Positive end-expiratory pressure
ROIs: Regions of interest
RM: Recruitment manoeuvres
SAPS2: Simplified Acute Physiology Score
SOFA: Sequential Organ Failure Assessment
T0: Baseline record
T1: Record at the end of the treatment
T2: Record after 1 h from the end of the treatment
T3: Record after 3 h from the end of the treatment oxygen saturation (SatO_2_)
TIV: Tidal impedance variation
