# Supplementary material for: Chest physiotherapy improves lung aeration in hypersecretive critically ill patients: a pilot randomized physiological study
Source: Crit Care. 2020 Aug 3;24:479. doi: 10.1186/s13054-020-03198-6 (PMC7396943; doi:10.1186/s13054-020-03198-6)
Supplement: Supplementary file 1 — Additional file 1: Table E1. Data from the 4 subgroups of patients stratified according the amount of secretions and application of recruiting manoeuvres. [file 13054_2020_3198_MOESM1_ESM.doc]

**­­­­­ONLINE SUPPLEMENTAL MATERIAL**

**­­­­­CHEST PHYSIOTHERAPY IMPROVES LUNG AERATION IN HYPERSECRETIVE CRITICALLY IL PATIENTS: A PILOT RANDOMIZED PHYSIOLOGICAL STUDY.**

Federico Longhini, MD^1^; Andrea Bruni, MD^1^; Eugenio Garofalo, MD^1^; Chiara Ronco, MD^2^; Andrea Gusmano, MD^2^; Gianmaria Cammarota, MD, PhD^3^; Laura Pasin, MD^4^; Pamela Frigerio, PT^5^; Davide Chiumello, MD, PhD^6,7,8^; Paolo Navalesi, MD, FERS^4,9^

**Affiliations:** ^1^Intensive Care Unit, University Hospital Mater Domini, Magna Graecia University, Catanzaro, Italy; ^2^Anesthesia and Intensive Care, Sant’Andrea Hospital, ASL VC, Vercelli, Italy; ^3^Department of Anesthesia and Intensive Care, "Maggiore della carità" University Hospital, Novara, Italy; ^4^Department of Anesthesia and Intensive Care, Ospedale Sant'Antonio, Padua, Italy; ^5^SC Anestesia e Rianimazione, Ospedale San Paolo – Polo Universitario, ASST Santi Paolo e Carlo, Milan, Italy^; 6^Dipartimento di Scienze della Salute, Università degli Studi di Milano, Milan, Italy; ^7^Centro ricerca coordinata di insufficienza respiratoria, Università degli Studi di Milano, Milan, Italy, ^9^Dipartimento di Medicina-DIMED, Università di Padova, Italy.

***Data acquisition and analysis***

Data were recorded by the EIT system at a sample of 20 Hz, coupled to data imported from the ventilator (*i.e.;* airway pressure, flow, tidal volume); afterwards, data were downloaded on a USB memory stick to be off line analyzed on a personal computer. Analysis was performed with dedicated software (EITdiag, Draeger Medical GmbH, Lübeck, Germany). The last three minutes of each record were analyzed. A "DATlowpass" filter was set at values higher than the patient's heart rate to purify the signal from the cardiac oscillation. EIT scans are represented by 32 × 32 color-coded matrix images showing impedance relative to the lowest recorded value (relative ΔZ) [1-2]. We measured the tidal impedance variation (TIV) as the difference between the relative ΔZ at the end of inspiration and expiration [1-2]. Tidal impedance changes were calibrated against known lung volume imported by the ventilator. Changes in TIV (ΔTIV, mL) and in end-expiratory lung impedance (ΔEELI, mL) were also computed [1-3]. We also defined two contiguous regions of interest (ROIs) of the same size (ventral and dorsal) and computed TIV, ΔTIV and ΔEELI for both [2-3]. The amount of pixel of non-ventilated area were also calculated and expressed as percentage of the pixel number. We also computed the centre of gravity (COG), which expresses the distribution of TIV in ventral to dorsal direction, calculated by dividing the dorsal by the overall TIV, expressed as percentage [4].

**Table E1. Data from the 4 subgroups of patients stratified according the amount of secretions and application of recruiting manoeuvres**

|  | **N RM- (n=15)** | | | |  | **N RM+ (n=15)** | | | |  | **H RM- (n=15)** | | | |  | **H RM+ (n=15)** | | | | ***P value*** |
| --- | --- | --- | --- | --- | --- | --- | --- | --- | --- | --- | --- | --- | --- | --- | --- | --- | --- | --- | --- | --- |
|  | ***T0*** | ***T1*** | ***T2*** | ***T3*** |  | ***T0*** | ***T1*** | ***T2*** | ***T3*** |  | ***T0*** | ***T1*** | ***T2*** | ***T3*** |  | ***T0*** | ***T1*** | ***T2*** | ***T3*** |  |
| Not ventilated area (%) | 0.7 [0.0; 3.6] | 0.7 [0.0; 3.6] | 0.7 [0.0; 1.6] | 0.7 [0.0; 4.0] |  | 0.2 [0.0; 0.8] | 0.0 [0.0: 1.0] | 0.0 [0.0; 0.0] | 0.0 [0.0; 0.6] |  | 0.2 [0.0; 2.1] | 1.4 [0.0; 4.6] | 0.4 [0.0; 1.9] | 0.9 [0.0; 7.1] |  | 0.7 [0.0; 4.4] | 1.0 [0.0; 4.2] | 1.4 [0.0; 7.7] | 1.4 [0.2; 4.4] | *0.101* |
| TIV (ml) | 436 [329; 470] | 435 [321; 495] | 427 [372; 474] | 442 [358; 541] |  | 459 [346; 609] | 468 [421; 724] | 477 [384; 578] | 478 [357; 578] |  | 459 [346; 609] | 468 [421; 724] | 477 [384; 578] | 478 [257; 578] |  | 464 [338; 498] | 362 [426; 513] | 468 [321; 515] | 416 [341; 491] | *0.564* |
| Dorsal | 177 [139; 245] | 178 [119; 250] | 201 [126; 221] | 187 [119; 258] |  | 196 [178; 257] | 218 [140; 289] | 211 [177; 255] | 211 [180; 246] |  | 154 [136; 214] | 147 [122; 203] | 152 [137; 185] | 138 [123; 161] |  | 177 [159; 245] | 185 [156; 236] | 182 [169; 242] | 202 [169; 224] | *0.105* |
| Ventral | 215 [172; 257] | 219 [174; 266] | 231 [185; 309] | 227 [202; 291] |  | 262 [168; 350] | 295 [202; 398] | 251 [175; 316] | 240 [165; 319] |  | 284 [209; 341] | 280 [212; 351] | 288 [170; 353] | 276 [194; 335] |  | 307 [213; 360] | 311 [202; 361] | 272 [197; 367] | 285 [204; 340] | 0.363 |
| ΔTIV (ml) | 0 [0; 0] | 6 [-41; 51] | 8 [-44; 92] | 14 [-20; 48] |  | 0 [0; 0] | 47 [-5; 85] | -12 [-79; 37] | 11 [-21; 30] |  | 0 [0; 0] | -5 [-42; 29] | -6 [-42; 31] | -18 [-54; -8] |  | 0 [0; 0] | -19 [-37; 1] | 4 [-46; 47] | -1 [41; 15] | 0.248 |
| Dorsal | 0 [0; 0] | 10 [-10; 22] | 8 [-40; 57] | 9 [-9; 36] |  | 0 [0; 0] | 3 [-14; 45] | 11 [-14; 45] | -11 [4; 27] |  | 0 [0; 0] | -1 [14; 10] | -2 [-7; 17] | -5 [-47; 19] |  | 0 [0; 0] | -9 [-18; 18] | 5 [-14; 34] | 5 [-8; 16] | *0.804* |
| Ventral | 0 [0; 0] | 4 [-31; 29] | 12 [-24; 32] | 20 [-22; 35] |  | 0 [0; 0] | 25 [4; 51] | -4 [-33; 25] | -2 [-24; 11] |  | 0 [0; 0] | 0 [-33; 18] | -8 [-23; 12] | -8 [-31; 13] |  | 0 [0; 0] | -10 [-27; 4] | -1 [-37; 13] | -10 [-20; 7] | *0.309* |
| Centre of gravity (%) | 51.8 [50.1; 55.1] | 52.2 [50.3; 55.2] | 51.8 [50.1; 56.3] | 51.9 [49.2; 55.2] |  | 51.9 [48.1; 53.9] | 52.3 [47.1; 54.2] | 51.2 [48.4; 54.2] | 51.0 [46.8; 53.5] |  | 55.0 [51.7; 57.9] | 55.0 [51.7; 58.5] | 54.5 [50.2; 58.4] | 55.0 [51.6; 58.7] |  | 54.6 [50.9; 57.0] | 54.9 [51.3; 56.6] | 54.6 [52.7; 55.9] | 54.3 [51.2; 55.9] | *0.124* |
| ΔEELI | 0 [0; 0] | -5 [-110; 87] | -129 [-165; 9] | -72 [-205; 9] |  | 0 [0; 0] | -24 [-171; 60] | -42 [-131; 69] | -102 [-214; 43] |  | 0 [0; 0] | 38 [-42; 271] | 138 [-47; 317] *,a | 105 [-43; 466] *,b |  | 0 [0; 0] | 63 [-59; 394] | 342 [207; 508] §,a | 144 [62; 449] §,b | *<0.001* |
| Dorsal | 0 [0; 0] | 2 [-45; 79] | -27 [-110; 115] | -52 [-157; 130] |  | 0 [0; 0] | 21 [-24; 74] | 6 [-55; 87] | 38 [-73; 82] |  | 0 [0; 0] | 57 [-12; 185] | 41 [-19; 500] *,a | 28 [-48; 414] *,b |  | 0 [0; 0] | 1 [-68; 293] | 150 [82; 408] §,a | 96 [20; 359] §,b | *0.002* |
| Ventral | 0 [0; 0] | -38 [-88; 19] | -66 [-227; -29] | -73 [-222; 7] |  | 0 [0; 0] | -59 [-139; 21] | -50 [-112; 20] | -37 [-140; 42] |  | 0 [0; 0] | -13 [-82; 57] | 54 [-16; 222] *,a | 5 [-91; 109] *,b |  | 0 [0; 0] | 83 [-9; 136] | 124 [44; 291] §,a | 115 [41; 268] §,b | *<0.001* |
| Heart rate (beat/min) | 78.6 (13.3) | 79.6 (17.3) | 79.4 (20.9) | 78.9 (19.2) |  | 88.6 (17.4) | 90.5 (16.6) | 89.2 (17.5) | 85.7 (16.3) |  | 80.1 (16.0) | 82.4 (15.1) | 79.5 (15.2) | 75.6 (13.6) |  | 79.0 (12.6) | 77.7 (13.2) | 74.0 (11.7) | 74.7 (13.0) | *0.489* |
| Mean Arterial Pressure (mmHg) | 85.1 (13.2) | 85.6 (11.0) | 84.2 (13.8) | 85.8 (11.9) |  | 86.5 (16.0) | 86.0 (17.9) | 85.0 (14.7) | 84.4 (15.4) |  | 82.4 (12.7) | 82.7 (13.0) | 81.4 (10.7) | 80.5 (10.8) |  | 92.7 (9.1) | 93.3 (10.6) | 88.2 (11.4) | 85.7 (10.3) | *0.414* |
| Respiratory Rate (breath/min) | 18.4 (4.3) | 18.3 (3.6) | 18.3 (4.1) | 18.3 (4.0) |  | 18.1 (3.6) | 18.0 (3.6) | 17.7 (3.7) | 18.7 (3.5) |  | 16.5 (5.8) | 16.7 (5.4) | 16.7 (5.9) | 16.5 (5.9) |  | 19.9 (5.5) | 20.5 (5.3) | 20.7 (5.1) | 20.0 (5.4) | *0.087* |
| pH | 7.43 (0.07) | 7.42 (0.06) | 7.43 (0.06) | 7.43 (0.06) |  | 7.43 (0.05) | 7.42 (0.06) | 7.43 (0.06) | 7.42 (0.06) |  | 7.41 (0.06) | 7.42 (0.07) | 7.42 (0.06) | 7.42 (0.06) |  | 7.42 (0.06) | 7.41 (0.05) | 7.42 (0.04) | 7.42 (0.04) | *0.618* |
| PaCO_2_ (mmHg) | 41.3 (9.9) | 42.5 (8.9) | 41.9 (9.4) | 42.1 (8.4) |  | 40.6 (6.5) | 41.9 (7.4) | 41.5 (6.9) | 41.5 (7.4) |  | 45.6 (6.8) | 44.3 (7.7) | 45.5 (7.7) | 45.4 (8.2) |  | 43.0 (10.1) | 44.2 (11.1) | 41.9 (8.8) | 41.5 (8.2) | *0.064* |
| PaO_2_/FiO_2_ (mmHg) | 255 (79) | 246 (65) | 257 (59) | 255 (59) |  | 222 (70) | 221 (65) | 224 (61) | 222 (58) |  | 226 (66) | 225 (68) | 227 (61) | 226 (63) |  | 211 (45) | 202 (43) | 213 (50) | 217 (49) | *0.619* |
| PEEP (cmH_2_O) | 6.3 (2.3) | | | |  | 8.1 (3.3) | | | |  | 6.9 (2.8) | | | |  | 7.2 (2.9) | | | | *0.376* |
| Mode of ventilation  (VC/PSV) | 3/12 | | | |  | 1/14 | | | |  | 2/13 | | | |  | 2/13 | | | | *0.778* |

a, p<0.05 T2 vs. T0 within the same subgroup; b, p<0.05 T3 vs. T0 within the same subgroup; * p<0.05 hypersecretive without RM (H RM-) vs. normosecretive without RM (N RM-); § p<0.05 hypersecretive with RM (H RM+) vs. normosecretive with RM (N RM+).

**SUPPLEMENTAL REFERENCES**

1. Bikker IG, Preis C, Egal M, Bakker J, Gommers D: Electrical impedance tomography measured at two thoracic levels can visualize the ventilation distribution changes at the bedside during a decremental positive end-expiratory lung pressure trial. *Crit Care* 2011, 15(4):R193.

2. Longhini F, Maugeri J, Andreoni C, Ronco C, Bruni A, Garofalo E, Pelaia C, Cavicchi C, Pintaudi S, Navalesi P: Electrical impedance tomography during spontaneous breathing trials and after extubation in critically ill patients at high risk for extubation failure: a multicenter observational study. *Ann Intensive Care* 2019, 9(1):88.

3. Mauri T, Eronia N, Abbruzzese C, Marcolin R, Coppadoro A, Spadaro S, Patroniti N, Bellani G, Pesenti A: Effects of Sigh on Regional Lung Strain and Ventilation Heterogeneity in Acute Respiratory Failure Patients Undergoing Assisted Mechanical Ventilation. *Crit Care Med* 2015, 43(9):1823-1831.

4. Luepschen H, Meier T, Grossherr M, Leibecke T, Karsten J, Leonhardt S: Protective ventilation using electrical impedance tomography. *Physiol Meas* 2007, 28(7):S247-260.
